# Literary Notes

**Published:** 1902-05

**Authors:** 


### LITERARY NOTES.

THE *Cleveland Medical Journal* is making a name for itself as one of the leading exponents of medical thought in the central area of the country. We have before called attention to the consolidation of two journals into this splendid magazine, and evidently no mistake was made in that union judging by the quality of the several editions of the new journal already issued.

THE Charity Eye and Ear and Throat Hospital, of Buffalo, has issued its 10th annual report for the year ending October 1, 1901. It makes a creditable showing of good work done. The death of Dr. F. W. Abbott, who was an active member of the staff, is touchingly recorded in it, accompanied by a plate portrait.

THE Interational Journal of Surgery Company has in press a new book by Dr. George G. Van Schaick, of New York, entitled, Regional Minor Surgery.

PRACTICE for sale. See advertising page xxiv.

